# Inflammation, underweight, malignancy and a marked catabolic state as predictors for worse outcomes in COVID-19 patients with moderate-to-severe disease admitted to Internal Medicine Unit

**DOI:** 10.1371/journal.pone.0268432

**Published:** 2022-05-18

**Authors:** Valeria Guglielmi, Luca Colangeli, Valeria Scipione, Simona Ballacci, Martina Di Stefano, Lauren Hauser, Michela Colella Bisogno, Monica D’Adamo, Emanuela Medda, Paolo Sbraccia

**Affiliations:** 1 Department of Systems Medicine, University of Rome Tor Vergata, Rome, Italy; 2 Obesity Medical Center, University Hospital Policlinico Tor Vergata, Rome, Italy; 3 Centre for Behavioural Sciences and Mental Health, Istituto Superiore Sanità, Rome, Italy; Heidelberg University Hospital, GERMANY

## Abstract

**Introduction:**

During COVID-19 pandemic, Internal Medicine Units (IMUs) accounted for about 70% of patients hospitalized. Although a large body of data has been published regarding the so-called first wave of the pandemic, little is known about the characteristics and predictors of worse outcomes of patients managed in IMUs during the second wave.

**Methods:**

We prospectively assessed demographics, comorbidities, treatment and outcomes, including ventilation support (VS) and death, in patients admitted to our IMU for SARS-CoV-2 between October 13th, 2020 and January 21st, 2021. Clinical evolution and biochemical testing 1, 7 and 14 days after COVID-19 diagnosis were recorded.

**Results:**

We studied 120 patients (M/F 56/64, age 71±14.5 years) admitted to our IMU. Most of them had at least one comorbidity (80%). Patients who died were older, more frequently underweight, affected by malignant neoplasms and on statin therapy compared to patients eventually discharged. Both worse outcome groups (VS and death) presented higher neutrophils, ferritin, IL-6 and lower total proteins levels than controls. Age was significantly associated with mortality but not with VS need. The multivariate analysis showed age and gender independent association of mortality with underweight, malignancy and antibiotics use at the admission. With regard to biochemical parameters, both unfavourable outcomes were positively associated with high WBC count, neutrophils, blood urea nitrogen and low serum total proteins.

**Conclusions:**

Our study identified inflammation, underweight, malignancy and a marked catabolic state as the main predictors for worse outcomes in COVID-19 patients admitted to IMU during the so-called second wave of the pandemic.

## Introduction

Since the beginning of the novel coronavirus disease 2019 (COVID-19) pandemic, in Italy there have been more than 15 million SARS-Coronavirus 2 (SARS‐CoV‐2) infected patients and more than 160.000 people died [1]. The overflow of COVID-19 patients has represented an enormous challenge for the Italian Public Health System. Internal Medicine Units (IMUs), that usually host patients with multiple chronic diseases presenting serious acute illnesses, played a fundamental role, accounting for about 70% of patients hospitalized [2–4]. In the context of the urgent need to develop effective therapeutic and preventive strategies and to define priority target groups for COVID-19 vaccines, identifying high-risk patients that may experience a more severe clinical presentation of the disease has quickly emerged as a main issue [5, 6].

Corradini et al. reported the results obtained from a nationwide registry of 3044 patients in 41 Italian medical wards during the period spanning from February to May 2020 (the so-called first wave of the pandemic), finding in age and number of pre-existing comorbidities the main factors associated with in-hospital death [7].

In this study, we prospectively aimed to investigate the association of demographic, clinical and biochemical characteristics and worse clinical outcomes in patients admitted to our IMU for SARS-CoV-2 during the second wave of the pandemic.

## Materials and methods

### Study design and patients

In this single-center prospective cohort study, we enrolled all (n = 120) consecutive patients admitted to our IMU for SARS‐CoV‐2, at the University Hospital “Fondazione PTV Policlinico Tor Vergata”, a tertiary health-care hospital in Rome (Italy), between October 13th, 2020 and January 21st, 2021, corresponding to the second wave of COVID-19 pandemic in Italy. All included patients had COVID‐19 diagnosis based on real-time reverse transcription-polymerase chain reaction (RT-PCR) nasopharyngeal swab and confirmed clinically/radiologically (i.e. as ground‐glass opacity and/or crazy paving on chest computed tomography scan). The study protocol was approved by the ethical committee of “Fondazione Policlinico Tor Vergata”. The informed consent to prospectively collect data from their medical records was obtained verbally by the patients in presence of two clinicians and one nurse and documented in the medical record.

### Data collection

Past medical history, treatments, clinical data and outcomes were collected for all consecutive COVID‐19 inpatients from their admission to our dedicated COVID‐19 IMU up to their discharge at home, Intensive Care Unit (ICU) transfer or death. This observational study was based on medical records, and patient confidentiality was protected by assigning an anonymous identification code and electronic data were stored in a password-protected computer. Collected data included clinical characteristics (age, sex and body mass index [BMI]), comorbidities, concomitant therapies and the date of the first nasopharyngeal swab sample testing positive for SARS‐CoV‐2 (referred to as day 0). Moreover, clinical evolution and biochemical testing during the hospital stay, namely 1, 7 and 14 days after COVID-19 diagnosis, were recorded.

BMI was calculated by patients recalled premorbid weight and height at admission. Grip strength was measured using a Camry digital hand dynamometer EH101 (Camry Scale, South El Monte, CA, United States). Low grip strength was based on cut-offs from Fried et al.’s original description [8], stratified by sex and BMI, but data were assessed only in a few patients so they are not reported. Blood pressure (BP) was recorded in sitting position with a standard, appropriately sized sphygmomanometer cuff (Perfect Aneroid, Erka, Germany). The average of two different readings was considered for the analysis. Biochemical tests (blood count, coagulation tests, glucose, HbA1c, lipid profile, liver and renal function parameters, electrolytes, albumin, prealbumin, creatine kinase, lactate dehydrogenase [LDH], high sensitivity C-reactive protein [hs-CRP], procalcitonin, interleukin [IL]-6 and tumor necrosis factor [TNF]-α) of blood samples obtained after overnight fast were assessed by routine laboratory techniques. Arterial blood gas analysis was performed using GEM® Premier™ 5000 (GP5000) blood gas analyzer (Instrumentation Laboratory, Bedford, MA, United States).

Non-neutralizing anti-SARS-CoV-2 IgG antibodies were detected in sera using immunofluorescent tests (Ichroma2™ COVID-19 Ab in conjunction with an Ichroma™ II Reader, Boditech Med Inc., South Korea).

### Study outcomes

The aim of this study was to evaluate the prevalence of comorbidities, concomitant therapies and biochemical abnormalities at the admission and during the hospital stay in patients with unfavourable outcomes, namely in patients who died during the hospital stay compared to the patients who were discharged, and in those who needed ventilation support (VS), both non-invasive mechanical ventilation (NIMV) and invasive mechanical ventilation (IMV), compared to those who did not need VS.

### Statistical analysis

Categorical variables were expressed as frequencies and percentage; Chi-squared and Fisher’s exact test were used to assess the significance of difference between groups. Continuous variables were presented as mean ± standard deviation and Mann-Whitney test was applied to assess differences between groups by unfavorable outcomes (VS need and death). As an approximation of the relative risk, crude odds ratio (OR) and related 95% confidence intervals (95% CI) for each single considered risk factor were calculated. Multivariate logistic regression was used to assess the association between different considered variables and dichotomous outcomes (VS [NIMV +/- IMV] and in-hospital mortality) taking into account the effect of age and gender. Continuous variables were categorized according to biological considerations or to clinical conventional cut-off points. An odds ratio was considered significant when 1.0 was not included in the 95% confidence interval.

Pearson correlation coefficient was estimated to test the degree of relationship between length of hospital stay and investigated biomarkers. In all statistical evaluations, a p value < 0.05 was considered as significant. Statistical analyses were conducted using STATA for Windows (version 16.0; StataCorp, College Station, TX, USA).

## Results

### Study population

**Fig 1:**
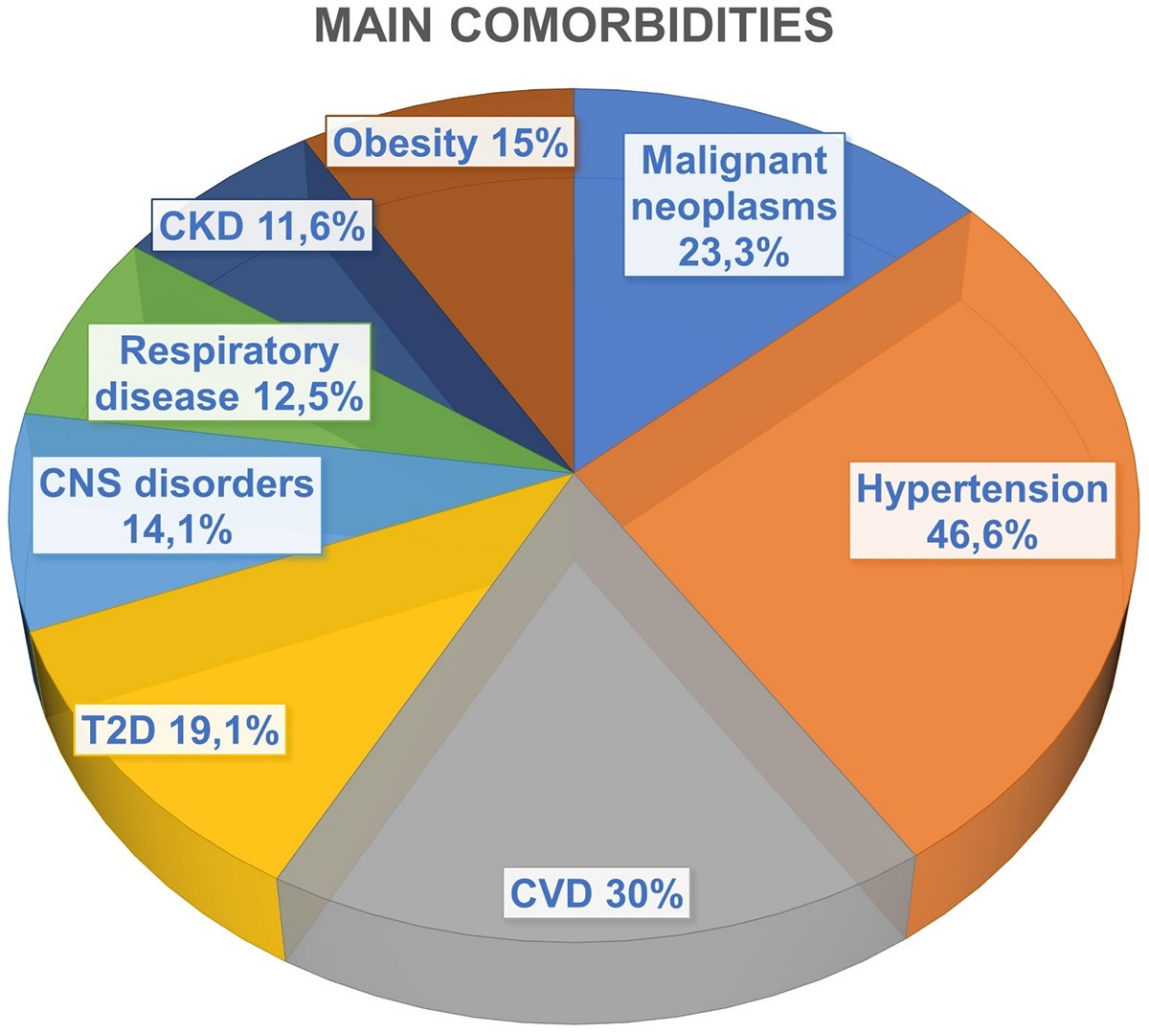
Main comorbidities of COVID-19 patients admitted in IMU. Abbreviations: CKD, chronic kidney disease; CNS, central nervous system, CVD, cardiovascular disease; T2D, type 2 diabetes.

We studied 120 consecutive patients admitted to our IMU. They were males in 46.6% of cases (n = 56), with mean age of 71.0±14.5 years, BMI 25.1±5.7 Kg/m^2^ and at least one comorbidity in 80% (n = 96) of cases. The prevalence of patients with comorbidities was 80%, 35.4% of them presented only one comorbidity, 31.3% two and 33.3% three or more. Main comorbidities were hypertension (46.6%), cardiovascular disease (30.0%), malignant neoplasms (23.3%), type 2 diabetes (T2D) (19.1%), obesity (15.0%), central nervous system (CNS) disorders (14.1%), respiratory diseases (12.5%) and chronic kidney disease (CKD) (11.6%) (Fig 1). Subjects with only one comorbidity were mainly affected by hypertension (22.9%) or cardiovascular disease (20.0%), whereas patients with two comorbidities showed hypertension and cardiovascular disease (16%) or hypertension and malignant neoplasms (16%).

Most frequently reported drugs at admission were angiotensin converting enzyme inhibitors (ACEi)/angiotensin II receptor blockers (ARB) (29.1%), diuretics (28.3%), acetylsalicylic acid (26.6%), beta-blockers (22.5%), statins (22.5%), oral anticoagulants (direct oral anticoagulants 9.1%; vitamin K antagonists 3.3%), metformin (10.8%), alcium-antagonists (6.0%).

During the study period, 61 patients (50.8%) needed oxygen therapy, 12 patients needed VS (10%) and 23 patients died (19.1%). Among the 12 patients who needed VS, 5 (4.1%) required IMV and were transferred to ICU; none of them died, so that all deaths occurred in IMU.

**Fig 2:**
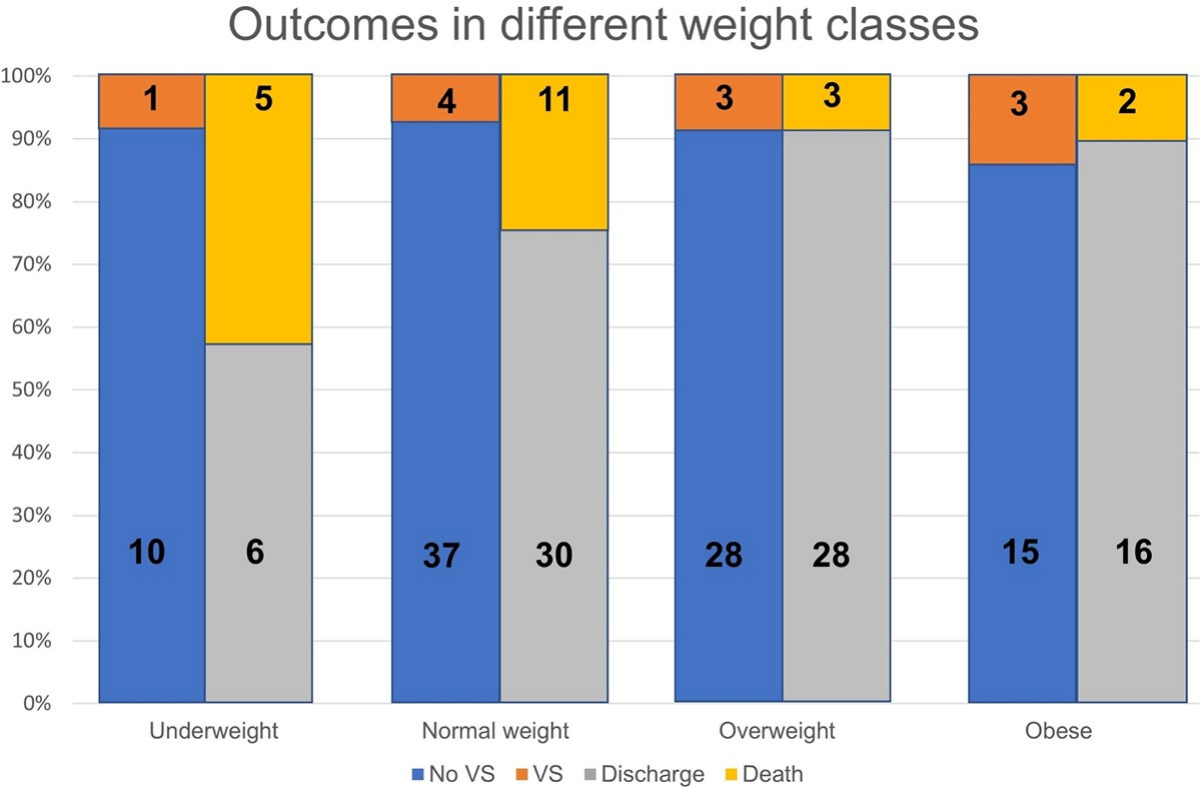
Outcomes among different BMI classes. Abbreviations: BMI, *body mass index*; VS, *ventilation support*. Numbers in bold indicate absolute number of patients.

BMI was registered for 101 patients: 11 patients were underweight (BMI < 18.5 kg/m^2^), 41 normal weight (BMI ≥ 18.5 and < 25 kg/m^2^), 31 overweight (BMI ≥ 25 and < 30 kg/m^2^) and 18 obese (BMI ≥ 30 kg/m^2^). Patients outcomes for BMI classes is shown in Fig 2: the highest incidence of death was evident in underweight group (5 of 11 patients, 45.5%).

### Patients outcome

#### Data and analyses 1 day after COVID-19 diagnosis

Baseline characteristics of patients who required VS (n = 12) and patients who died (n = 23) during the hospital stay compared to those who did not are presented in Table 1.

**Table 1:** Baseline characteristics of COVID-19 patients who presented unfavorable outcomes (ventilation support need and death) and those who did not.

	No VS	VS	*p value*	Discharge	Death	*p value*
	(n = 106)	(n = 12)		(n = 96)	(n = 23)	
	Mean ± SD or %	Mean ± SD or %		Mean ± SD or %	Mean ± SD or %	
**Males (%)**	44.3	66.7	*ns*	44.8	52.2	*ns*
**Age (y)**	71.9 ± 14.6	64.8 ± 13.6	*0*.*08*	69.6 ± 14.4	77.5 ± 13.1	*0*.*01*
***COMORBIDITIES***						
**T2D (%)**	21.7	0	*nc*	19.8	17.4	*ns*
**Hypertension (%)**	47.2	41.7	*ns*	46.9	47.8	*ns*
**Underweight (%)**	11.3	8.3	*ns*	6.3	27.2	*0*.*005*
**Overweight (%)**	31.8	25	*ns*	36.7	13.6	*0*.*005*
**Obese (%)**	17.1	25	*ns*	20.3	9.1	*0*.*01*
**CVD (%)**	29.3	41.7	*ns*	29.1	34.8	*ns*
**Respiratory diseases (%)**	14.1	0	*nc*	12.5	13.0	*ns*
**CNS Disease (%)**	16.0	0	*nc*	11.5	26.1	*0*.*07*
**Malignancy (%)**	23.6	25.0	*ns*	18.8	43.5	*0*.*01*
**CKD (%)**	12.8	8.3	*ns*	9.4	21.7	*0*.*09*
***CONCOMITANT THERAPIES***						
**Metformin (%)**	13.9	0	*nc*	13.1	11.7	*ns*
**Insulin (%)**	1.1	0	*nc*	1.2	0	*ns*
**Acetyilsalicylic acid (%)**	32.3	25.0	*ns*	31.0	33.3	*ns*
**Oral anticoagulants (%)**	16.1	0	*nc*	14.3	16.7	*ns*
**Beta-blockers (%)**	26.9	28.6	*ns*	26.2	29.4	*ns*
**ACEi/ARB (%)**	34.4	42.9	*ns*	36.9	23.5	*ns*
**Diuretics (%)**	35.5	14.3	*ns*	33.3	35.3	*ns*
**Statins (%)**	25.8	28.6	*ns*	22.6	47.1	*0*.*03*

Abbreviations: ACEi, *angiotensin converting enzyme inhibitor*; ARB, *angiotensin receptor blocker*; CKD, *chronic kidney disease*; CNS, *central nervous system*; CVD, *cardiovascular disease*; ns, *not significant*; SD, *standard deviation*; T2D, *type 2 diabetes*; VS, *ventilation support*.

Patients who died resulted older, more frequently underweight, affected by malignant neoplasms and on previous statin therapy compared to patients eventually discharged from the hospital. They had a more severe pulmonary involvement (lower PaO_2_/FiO_2_) and we additionally observed a trend toward a higher frequency of CNS disorders (p = 0.07), CKD (p = 0.09) and antibiotics prescription at admission (p = 0.06). Patients who needed VS did not differ from relative controls in terms of comorbidities and concomitant therapies.

Baseline clinical parameters are shown in Table 2. Both worse outcome groups (VS and death) presented higher inflammation (neutrophil count and percentage, ferritin and IL-6), triglycerides levels and lower serum total proteins as compared to controls. Patients who died also presented lower hemoglobin (Hb, p = 0.05), higher white blood cells (WBCs), blood urea nitrogen (BUN), uric acid, NT-proBNP, and lower albumin and 25OH-vitamin D levels.

**Table 2:** Baseline clinical parameters of COVID-19 patients who presented unfavorable outcomes (ventilation support need and death) and those who did not.

	No VS	VS	*p value*	Discharge	Death	*p value*
	(n = 106)	(n = 12)		(n = 96)	(n = 23)	
	Mean ± SD or %	Mean ± SD or %		Mean ± SD or %	Mean ± SD or %	
***HOSPITALIZATION GENERALITIES***						
**Onset of symptoms (days)**	4.4 ± 3.8	5.0 ± 2.7	*ns*	4.6±3.8	2.3±1.0	*ns*
**Lenght of hospital stay (days)**	12.8 ± 11.9	21.7 ± 13.4	*0*.*06*	13.4 ± 12.7	14.4 ± 10.5	*ns*
**Oxygen therapy (%)**	17.9	25.0	*ns*	15.3	30.4	*ns*
**Antibiotic therapy (%)**	24.5	25.0	*ns*	20.8	39.1	*0*.*06*
**Remdesivir therapy (%)**	38.9	58.3	*ns*	44.4	27.3	*ns*
**Dexamethasone therapy (%)**	38.9	71.4	*ns*	71.0	66.6	*ns*
**Heparin therapy (%)**	94.8	87.5	*ns*	94.2	95.2	*ns*
***CLINICAL PARAMETERS AT ADMISSION***						
**BMI (Kg/m** ^**2**^ **)**	24.9 ± 5.2	26.9 ± 8.7	*ns*	25.8 ± 4.8	22.7 ± 7.2	*0*.*001*
**SBP (mmHg)**	130.4 ± 23.4	135.0 ± 14.9	*ns*	132.5 ± 20.4	124.9 ± 16.6	*ns*
**DBP (mmHg)**	72.6 ± 11.4	71.9 ± 13.4	*ns*	73.7 ± 11.2	69.6 ± 12.3	*ns*
**Heart rate (beats/min)**	80.7 ± 14.4	88.9 ± 12.7	*ns*	80.2 ± 15.0	85.3 ± 11.8	*ns*
**PaO**_**2**_ **/FiO**_**2**_	331.7±97.5	275.8±65.8	*0*.*08*	344.4 ± 95.4	272.7 ± 77.5	*0*.*009*
***BIOCHEMICAL PARAMETERS AT ADMISSION***						
**Hb (g/dL)**	12.2 ± 2.4	12.0 ± 2.6	*ns*	12.4 ± 2.5	11.2 ± 2.0	*0*.*05*
**Platelets (x10^9/L)**	250.4 ± 160.9	200.1 ± 107.5	*ns*	255.5 ± 160.7	212.9 ± 131.0	*ns*
**WBCs (x10^9/L)**	10.6 ± 14.7	12.4 ± 7.2	*ns*	9.64 ± 15.01	14.3 ± 8.9	*0*.*001*
**Neutrophils (x10^9/L)**	6.9±5.9	10±6	*0*.*1*	5.6 ± 3.8	12.4 ± 8.1	*0*.*0001*
**Lymphocytes (x10^9/L)**	1.3 ± 0.8	1.6 ± 1.4	*ns*	1.5 ± 0.9	1.0 ± 0.6	*0*.*08*
**D-dimer (ng/mL)**	1584.9 ± 1575.4	29464 ± 64757	*ns*	5342.5 ± 24745	3328.8 ± 2549.9	*ns*
**hs-CRP (mg/L)**	68.0 ± 66.0	67.0 ± 56.4	*ns*	61.4 ± 62.9	83.4 ± 67.1	*ns*
**Procalcitonin (ng/mL)**	1.0 ± 2.0	0.1 ± 0.1	*ns*	0.7 ± 1.8	1.1 ± 1.8	*ns*
**LDH (UI/L)**	390.4 ± 394.5	404.8 ± 184.06	*ns*	307.7 ± 129.3	626.0 ± 642.1	*ns*
**AST (UI/L)**	99.2 ± 213.6	36.2 ± 18.5	*ns*	53.7 ± 61.9	213.4 ± 365.0	*ns*
**ALT (UI/L)**	50.0 ± 71.4	33.1 ± 26.6	*ns*	46.6 ± 65.7	56.8 ± 74.9	*ns*
**CK (UI/L)**	204.1 ± 460.9	182.9 ± 204.3	*ns*	430.3 ± 841.1	128.8 ± 130.9	*ns*
**Creatinine (mg/dL)**	1.28 ± 1.11	0.95 ± 0.40	*ns*	1.18 ± 1.11	1.44 ± 0.94	*ns*
**BUN (mg/dL)**	62.1 ± 53.0	43.6 ± 15.8	*ns*	48.2 ± 41.2	92.0 ± 61.0	*0*.*0001*
**Sodium (mmol/L)**	139.1 ± 6.6	136.2 ± 3.9	*ns*	138.6 ± 4.3	139.6 ± 10.7	*ns*
**Potassium (mmol/L)**	4.1 ± 0.6	3.9 ± 0.5	*ns*	4 ± 0.5	4.4 ± 0.7	*0*.*08*
**Chlorides (mmol/L)**	103.9 ± 6.5	101.1 ± 3.9	*ns*	103.5 ± 4.6	104 ± 10	*ns*
**Calcium (mg/dL)**	8.5 ± 0.7	8.8 ± 1	*ns*	8.5 ± 0.6	8.7 ± 1	*ns*
**Albumin (g/L)**	3.6 ± 0.6	3.7 ± 0.6	*ns*	3.8 ± 0.5	3.1 ± 0.6	*0*.*0002*
**Total proteins (g/L)**	6.0 ± 0.8	5.2 ± 0.9	*0*.*02*	6.1 ± 0.8	5.4 ± 1.0	*0*.*003*
**Uric acid (mg/dL)**	5.4 ± 2.6	5.4 ± 1.8	*ns*	5.1 ± 2.1	6.8 ± 3.4	*0*.*01*
**25(OH) Vit D (ng/mL)**	17.9 ± 13.8	15.7 ± 8.6	*ns*	19.4 ± 14.0	11.7 ± 8.2	*0*.*02*
**Triglycerides (mg/dL)**	145.2±62.7	203.3±91.9	*0*.*04*	141.8 ± 61.1	195.5 ± 80.6	*0*.*006*
**Ferritin (ng/mL)**	859.7±1130	2514.5±3140	*0*.*02*	832.0 ± 1200.8	1854.8 ± 2282.5	*0*.*005*
**NT-proBNP (pg/mL)**	182.9 ± 321.8	198.1 ± 362.9	*ns*	128.5 ± 190.0	382.6 ± 561.2	*0*.*001*
**HbA1c (mmol/L)**	43.8 ± 12.6	42.5 ± 7.9	*ns*	43.0 ± 12.1	45.7 ± 12.5	*ns*
**IL-6 (pg/mL)**	7.4 ± 5.1	59.1±95.2	*0*.*02*	23.3 ± 27.3	49.4 ± 69.7	*0*.*05*
**TNF-α (pg/mL)**	15.0 ± 13.9	31.9 ± 33.1	*ns*	15.5 ± 16.1	28.5 ± 26.5	*ns*

Abbreviations: ALT, *alanine aminotransferase*; AST, *aspartate aminotransferase*; BUN, *blood urea nitrogen*; CK, *creatin kinase*; Hb, *hemoglobin*; hsCRP, *high sensitivity C-reactive protein*; HbA1c, *glycated hemoglobin*; IL-6, *interleukine-6*; LDH, *lactate dehydrogenase*; MCV, *mean corpuscolar volume*; ns, *not significant*; NT-proBNP, *N-terminal fragment of the prohormone brain-type natriuretic peptide*; RBCs, *red blood cells*; SD, *standard deviation*; TNF-α, *tumor necrosis factor-α*; VS, *ventilation support;* WBCs, *white blood cells*.

Age was significantly associated with mortality (OR = 1.04, 95% CI 1.01–1.08; p = 0.02), but not with VS need (OR = 1.0, 95% CI 0.93–1.00; p = 0.11).

The multivariate analysis confirmed a positive age and gender independent association between COVID-19 mortality and underweight and malignancy. On the contrary, fatalities showed a trend toward a negative association with overweight (p = 0.07) but not with obesity (Table 3).

**Table 3:** Age- and gender-adjusted odds ratios of associations between comorbidities and ventilation support or in-hospital mortality.

	VS (NIMV +/- IMV)		In-hospital mortality	
**Comorbidities**	**OR**_**adj**_ **(95% CI)**	***p value***	**OR**_**adj**_ **(95% CI)**	***p value***
**T2D**	–	*nc*	0.8 (0.2–2.6)	*ns*
**Hypertension**	1.0 (0.3–3.8)	*ns*	0.7 (0.3–1.8)	*ns*
**Underweight**	1.1 (0.1–10)	*ns*	5.0 (1.2–20.8)	*0*.*02*
**Overweight**	0.7 (0.2–3.1)	*ns*	0.3 (0.1–1.1)	*0*.*07*
**Obesity**	1.6 (0.3–6.9)	*ns*	0.5 (0.1–2.4)	*ns*
**CVD**	2.3 (0.6–8.9)	*ns*	1.0 (0.4–2.7)	*ns*
**Respiratory diseases**	–	*nc*	0.9 (0.2–3.6)	*ns*
**CNS diseases**	–	*nc*	1.6 (0.45–5.7)	*ns*
**Malignancy**	1.1 (0.3–4.7)	*ns*	4.1 (1.5–11.8)	*0*.*008*
**CKD**	0.8 (0.1–7.3)	*ns*	2.0 (0.6–7.0)	*ns*

Abbreviations: CKD, *chronic kidney disease*; CNS, *central nervous system*; CVD, *cardiovascular disease*; IMV, *invasive mechanical ventilation*; nc, *not calculable*; NIMV, *non-invasive mechanical ventilation*; ns, *not significant*; T2D, *type 2 diabetes*; VS, *ventilation support*. *OR*_**adj**_, *age- and gender-adjusted Odds Ratio; 95%CI*, *95% Confidence Intervals*.

We did not detect significant difference in outcome in patients treated with dexamethasone, heparin and the antiviral drug remdesivir. Only the use of antibiotics at the admission resulted independently associated to mortality (OR 3.1, 95% CI 1.1–8.7; p = 0.03).

With regard to biochemical parameters, at multivariate analysis we found a positive association of VS need with high WBC count and low serum total proteins. We also observed a trend toward a positive association of VS need and high IL-6 levels (Table 4).

**Table 4:** Age—and gender-adjusted odds ratios of significant associations between biochemical parameters assessed at day 1 and unfavorable outcomes (ventilation support and death).

	VS (NIMV +/- IMV)		In-hospital mortality	
	**OR**_**adj**_ **(95% CI)**	***p value***	**OR**_**adj**_ **(95% CI)**	***p value***
**Low Hb**	1.9 (0.4–9.2)	*ns*	4.9 (1.4–17.6)	*0*.*02*
**High WBC**	5.9 (1.0–32.7)	*0*.*04*	7.1 (1.9–26.3)	*0*.*003*
**High neutrophils**	3.1 (0.7–13.9)	*ns*	21.5 (4.1–111.7)	*0*.*0001*
**High BUN**	0.3 (0.03–2.7)	*ns*	5.1 (1.6–16.5)	*0*.*007*
**Low albumin**	1.3 (0.1–14.0)	*ns*	5.7 (1.2–27.1)	*0*.*03*
**Low total proteins**	12.0 (1.7–83.3)	*0*.*01*	3.6 (1.1–11.6)	*0*.*03*
**High IL-6**	6.7 (0.8–57.4)	*0*.*08*	1.6 (0.2–11.0)	*ns*
**Low PaO**_**2**_ **/FiO**_**2**_	2.3 (0.7–8.4)	*ns*	2.4 (0.9–6.3)	*ns*

Abbreviations: BUN, *blood urea nitrogen*; Hb, *hemoglobin*; IL-6, *interleukin-6*; IMV, *invasive mechanical ventilation*; NIMV, *non-invasive mechanical ventilation*; ns, *not significant*; VS, *ventilation support*; WBC, *white blood cell*. Low Hb if < 12 g/dL; High WBC > 10.8 x10^9^/L; High neutrophils > 7.2 x10^9^/L; High BUN > 55 mg/dL; Low albumin < 3 g/L; Low total proteins < 6 g/L; High IL-6 > 12.4 pg/mL; Low PaO_2_/FiO_2_ < 400. *OR*_**adj**_, *age- and gender-adjusted Odds Ratio; 95%CI*, *95% Confidence Intervals*

This analysis also confirmed the association between COVID-19 mortality and low Hb concentration, high WBC count, high neutrophils, low albumin, low total proteins and high BUN (Table 4).

#### Data and analyses 7 days after COVID-19 diagnosis

After 7 days from the first positive swab, both groups (VS and death) presented greater respiratory disease severity (lower P/F ratio) (VS: 215.5±103, no VS: 297.2±76, p = 0.07; death: 245±91, discharge: 300±78, p = 0.04) and were characterized by higher hs-CRP levels (VS: 81.4±52.3 mg/L, no VS: 46.2±65 mg/L, p = 0.004; death: 83.9±67.6 mg/L, discharge: 41.9±60 mg/L, p = 0.002) and neutrophil percentage (VS: 84.1±8.5%, no VS: 74.3±16%, p = 0.01; death: 81.7±22%, discharge: 73.7±12.6%, p = 0.0007) compared to their respective controls. Only in the group of patients who died, higher levels BUN (death: 106.4±70.3 md/dL, discharge: 48.2±29 mg/dL, p = 0.0002) and decreased albumin (death: 2.8±0.4 g/L, discharge: 3.3±0.3 g/L, p = 0.0004) and Hb (death: 10.3±2.2 g/dL, discharge: 11.9±2.2 g/dL, p = 0.01) were observed as compared to discharged patients.

Multivariate analysis (age- and gender-adjusted) of data collected at day 7 are shown in Table 5. High WBC count, high neutrophils and high BUN measured at day 7 were positively associated with both worse outcomes (VS and death).

**Table 5:** Significant age- and gender-adjusted associations of biochemical parameters assessed at day 7 with unfavorable outcomes (VS and death).

	VS (NIMV +/- IMV)		In-hospital mortality	
	**OR**_**adj**_ **(95% CI)**	***p value***	**OR**_**adj**_ **(95% CI)**	***p value***
**Low Hb**	1.5 (0.4–5.8)	*ns*	3.6 (0.9–13.6)	*0*.*05*
**High WBCs**	9.6 (1.6–57.0)	*0*.*01*	5.6 (1.4–22.2)	*0*.*02*
**High neutrophils**	11.5 (1.3–105.2)	*0*.*03*	12.4 (1.5–101.5)	*0*.*02*
**High BUN**	5.0 (1.0–24.4)	*0*.*04*	4.2 (1.2–14.8)	*0*.*03*
**Low albumin**	0.8 (0.1–4.5)	*ns*	27.0 (4.8–151.0)	*0*.*0001*
**Low PaO**_**2**_ **/FiO**_**2**_	5.8 (1.3–25.6)	*0*.*02*	1.0 (0.4–3.0)	*ns*

Abbreviations: BUN, *blood urea nitrogen*; Hb, *hemoglobin*; IMV, *invasive mechanical ventilation*; nc, *not calculable*; NIMV, non-invasive mechanical ventilation; ns, *not significant*; VS, *ventilation support*; WBCs, *white blood cells*. Low Hb if < 12 g/dL; High WBC > 10.8 x10^9^/L; High neutrophils > 7.2 x10^9^/L; High BUN > 55 mg/dL; Low albumin < 3 g/L; Low PaO_2_/FiO_2_ < 400. *OR*_**adj**_, *age- and gender-adjusted Odds Ratio; 95%CI*, *95% Confidence Intervals*.

The analysis confirmed the association between COVID-19 in-hospital mortality and low Hb, high WBC, high neutrophils, high BUN and low albumin levels.

At day 7, a new association between high BUN with VS need, not present at admission, became also evident.

Anti-SARS-CoV-2 IgG antibodies were collected on day 7, 14 and, for those patients still hospitalized, 21 after the first nasopharyngeal swab samples testing positive for SARS‐CoV‐2. As predictable, antibodies level became higher at day 14, but there was no significant association with the outcome (Table 6).

**Table 6:** Anti-SARS-CoV-2 IgG antibodies levels in patients who presented unfavorable outcomes (VS and death) and in those who did not.

	No VS (n = 106)	VS (n = 12)	*p value*	Discharge (n = 96)	Death (n = 23)	*p value*
**Anti-SARS-CoV-2 IgG (day 7) (U/ml)**	2.4 ± 2.5	4.8 ± 0.0	*ns*	2.8 ± 2.6	1.6 ± 1.9	*ns*
**Anti-SARS-CoV-2 IgG (day 14) (U/ml)**	4.7 ± 2.2	4.1 ± 1.8	*ns*	4.3 ± 2.1	4.8 ± 2.3	*ns*
**Anti-SARS-CoV-2 IgG (day 21) (U/ml)**	4.9 ± 1.9	4.2 ± 3.6	*ns*	5.4 ± 1.6	3.5 ± 2.8	*ns*

Abbreviations: ns, *not significant*; VS, *ventilation support*.

## Discussion

In this single-center prospective cohort study, we enrolled 120 (M/F: 56/64) consecutive patients admitted to our IMU for SARS‐CoV‐2 during the so-called second wave of the pandemic. At admission, patients were considered to have moderate-to-severe disease. Indeed, critically ill cases, defined by respiratory failure requiring mechanical ventilation, septic shock, disseminated coagulopathy or other organs failure [9], were directed to ICUs.

Patients hospitalized in IMUs are usually old and with many comorbidities [10]. Also during COVID-19 pandemic the mean age of patients admitted in our IMU was 71.0±14.5 years and we confirm that older age is one of the main predictor of in-hospital mortality [11–14]. The majority (80%; n = 96) of cases presented with at least one comorbidity. Hypertension, which has been identified as an independent risk factor for COVID-19 mortality [15], was the most common comorbidity (46.6%). However, in our cohort malignancy and underweight, but not hypertension, resulted independent predictors of *exitus*.

With regard to BMI, since the beginning of the pandemic obesity emerged as one of the main independent risk factors for developing severe forms of COVID-19 [16]. However, we found no statistical association between obesity and mortality. Rather, in our frail population, underweight entailed a 5-fold increased risk of worse outcome compared to normal weight subjects in line with previous reports [17], while overweight showed a trend toward a negative association with fatalities. Substantially, our findings partly tend to replicate the j-shaped curve association showed by Min Gao et al. in a large cohort study in England [18]. These authors described an increased risk of death for people with BMI ≤ 20 kg/m^2^ and ≥ 28 kg/m^2^. However, in the same study the association between increasing BMI and admission to ICU was linear. But we should consider that ICU admission of patients with pre-existing frailty is usually discouraged and unlikely during the COVID-19 pandemic (due to the dramatic overflow of patients) and this explains the high prevalence of cachectic and neoplastic patients in IMUs, as we found in our study.

The identification of alert signs is crucial in such a multifaceted disease as COVID-19 and many authors have tried to validate simple scores for the prediction of unfavourable outcomes [19]. Among laboratory tests at admission, neutrophil/lymphocyte ratio, CRP and D-dimer have been the most used in defining disease severity [20]. We not only confirmed the predictive value of high WBC count and neutrophilia for COVID-19 mortality and VS need, but in patients with unfavourable outcomes we also found increased levels of two other markers of inflammation rather than CRP: ferritin and IL-6. Ferritin, an iron storage protein whose role in a multitude of other conditions, including inflammatory and malignant diseases, is increasingly recognized [21], was found elevated in individuals with severe COVID-19 [22]. In this context, it is not surprising that even high IL-6 serum concentrations have been correlated to disease COVID-19 severity [23]. After all, the inflammatory cytokine storm, an excessive and uncontrolled release of pro-inflammatory cytokines resulting in lungs and other organs damage, has been recognized as the primary cause of death in COVID-19 [24]. This is the reason why IL-6 inhibitors (tocilizumab, siltuximab and sarilumab) had been proposed as potential therapies in critical COVID-19 patients [25]. Substantially, we can assume that higher inflammation at admission (especially in terms of WBC count, neutrophil percentage, ferritin and IL-6) corresponds to higher risk for worse outcomes.

Both VS need and in-hospital mortality were found associated to low total proteins and high BUN levels.

Even though BUN is widely used as endogenous filtration marker for evaluation of kidney function and blood volume, it is also used in CURB-65, a severity scoring system for community-acquired pneumonia patients [26]. In this view, high BUN levels at admission have been found robustly associated with mortality in critically ill patients admitted to ICU [27] independent of renal failure [28]. Also hypoproteinemia is predictive for COVID-19 outcomes [29]. Given that BUN is a nitrogenous end product of protein metabolism, the elevation of this parameter together with the presence of low serum total proteins may suggest the presence of a disturbed protein metabolism and neurohumoral activation [30] which is, as said, associated to severe forms of COVID-19 [31], possibly indicating a marked catabolic state.

Predictors of in-hospital mortality assessed at admission and at day 7 did not vary.

Instead, for what concerns predictors of the need for VS, high WBC and low serum total proteins should be assessed at admission in order to identify patients at greater risk of developing a more severe respiratory disease, while high neutrophils, high BUN and low PaO_2_/FiO_2_ gain predictive value at day 7. Accordingly, Liu et al. found that dynamic changes in BUN were associated with adverse outcomes in COVID-19 more than the baseline parameters [14].

As highlighted in previous studies [32], clinicians should be aware of the potential for some COVID-19 patients to deteriorate rapidly, about one week after symptoms onset. So it is not only crucial to follow strictly the patients by arterial blood gas analysis in order to detect and prevent hypoxemic events, but also to assess other biochemical parameters in the proper timing to foresee the disease evolution. Therefore, we believe that longitudinal monitoring of WBCs, neutrophils count, BUN and PaO_2_/FiO_2_ during hospitalization may help to identify severe patients and predict the progression of COVID-19 toward worse outcomes.

This study has some limitations. First of all, severely or critically ill patients with COVID-19 usually are admitted in ICUs and not in IMUs, so that the case fatality rate in this study cannot reflect the true mortality of COVID-19 and there is a selection bias for prognostic generalizations. Secondly, for practical reasons (e.g. patient isolation) weight and height reported in results were not measured but recalled by the patients. Finally, this is a single-center study with a limited sample size and some parameters (for example, hand grip strength) were collected in few patients so that they didn’t have sufficient statistical power to draw conclusions.

## Conclusions

In this study we presented our experience as IMU involved in COVID-19 patients management during the so-called second phase of pandemic in Italy. Patients hospitalized for COVID-19 in IMUs are usually patients that do not need VS, at least at the admission, but they are generally older and with comorbidities, so that they are more fragile and inclined to deterioration during the hospital stay. In particular, we found that older age, inflammation, underweight, malignancy and a marked catabolic state are the main predictors for a worse outcome in COVID-19 patients admitted to IMU. Early evaluation of WBCs, neutrophils, BUN, total proteins and careful follow-up of white blood cells, neutrophils, BUN and PaO_2_/FiO_2_ can identify patients at risk of death or VS need.
